# Evaluating the Impact of a Paediatric Burn Club for Children and Families Using Group Concept Mapping

**DOI:** 10.3390/ebj4020019

**Published:** 2023-05-10

**Authors:** Emma L. Hodgkinson, Alison McKenzie, Louise Johnson

**Affiliations:** Northern Regional Burns Service, Newcastle upon Tyne Hospitals NHS Foundation Trust, Newcastle upon Tyne NE1 4LP, UK

**Keywords:** burn care, burn camps, group concept mapping, burn rehabilitation

## Abstract

Access to burn camps and clubs is cited as an essential element for children following a burn injury. In the Northern Regional Burns Service, this takes the form of a club run by a multidisciplinary team, offering residential camps and family day trips. In this service evaluation, Group Concept Mapping was used to evaluate the perception of the club by staff, children and families. Opportunistic sampling was used to seek responses to the following prompts: “The challenges for children and families after a burn injury are…” and “The role of The Grafters Club is…”. The results indicate that participants perceived the club to be effective at addressing body image and confidence issues for the children but highlighted an unmet parental expectation that the club would also facilitate the sharing of experiences, normalisation of emotional reactions, and processing of guilt and other psychological distress for parents. When taken concurrently with pre-existing evidence in the literature base, it is proposed that a club model of psychosocial support for children and families could provide an accessible and informal opportunity for parental support that may be less subject to barriers perceived with traditional formal psychological support.

## 1. Introduction

The benefits of burn camps for children with a burn injury are well documented worldwide [1] and are recommended as an essential element of routine care following a burn injury within the National Burn Care Standards in the UK [2]. The impact and effectiveness of camps has been well established within the literature base; attendance at a burn camp has been demonstrated to promote confidence building and psychological recovery [1], satisfaction with appearance and social concerns [3], a sense of belonging or normalisation [4], new relationships and a sense of achievement [5]. Despite these definitive benefits identified in qualitative studies, they do not appear to be adequately captured by traditional quantitative methods causing an incongruence in the evidence base [6].

Traditionally burn camps are targeted at the child themselves. However, the evidence in the literature highlights the impact on the wider family network, with studies describing long-term issues with guilt and post-traumatic stress reactions [7]. In their qualitative study, Heath et al. [8] reported parents describing both physical and psychological isolation during and after their child’s acute burn treatment noting that, while psychosocial support was known to be available, there were significant barriers to accessing it, particularly during outpatient treatment. This study also highlighted that parents desired to speak with parents who had been through a similar experience, reflecting that this reduced the sense of isolation as well as providing strategies to manage specific issues. Other studies have similarly described the role and benefits of peer support and effective mutual aid in healthcare settings, e.g., [9], but suggest that there are gaps in this type of service provision in the UK due to recruitment and attendance issues [10].

Within the Northern Regional Burns Service, both burn camps for children and peer support for parents sit within the provision of a dedicated burn club (“The Grafters Club”) for children and families following a burn injury that either requires grafting or input from the scar management service following healing. All children are invited to join the club once their wounds are healed and receive twice-yearly newsletters plus invitations to residential burn camps, family day trips (i.e., to theme parks) and family parties. The club is managed within the broader hospital charity, and fundraising is entirely undertaken by staff, members of the club and interested donors. Activities are supported by volunteers and staff from the multidisciplinary team. While regular evaluation is undertaken following every event, it was decided to undertake an evaluation exploring the role of the club as a community (rather than individual events) from the perspective of staff and families as well as the children themselves. This was particularly felt to be important on the recommencement of usual club activities following the restrictions during the COVID-19 pandemic, where events were either cancelled or held virtually, e.g., [11].

Group concept mapping (or structured conceptualisation [12]) integrates familiar group processes such as brainstorming and idea sorting with multivariate statistical analysis to produce a picture or map [13]. Concept mapping has been identified to be a useful tool in healthcare settings due to its integration of input from multiple and differing sources, rigorous statistical analysis, and visual depiction of the group’s thinking that can be used to guide service development and improvement [13]. To undertake concept mapping, ideas are generated through brainstorming amongst a group of participants around a specified focus before being synthesised, sorted and rated for variables such as importance or feasibility. The resulting data are subjected to statistical analysis to yield a visual map. In the current context, concept mapping was considered to offer advantages over other methods. For example, it would permit the exploration and integration of ideas from the diverse stakeholders in The Grafters Club (staff, children and families) and would be feasible to implement with an opportunistic sample at a club event. Furthermore, the resulting map could offer clear guidance for additional evaluation, service development and improvement. 

### Aims

The aims of the current evaluation are to evaluate the perception of the Northern Regional Burns Service’s club for children and families (The Grafters Club) using group concept mapping both to support service planning in this club and to make recommendations for burn club provision across other services. 

## 2. Materials and Methods

### 2.1. Participants

Opportunistic sampling was undertaken during the 2022 Grafters Club Christmas party. The attendees were given a verbal description and written information sheet about the evaluation and invited to participant. One hundred and twenty children and adults from thirty families attended the party, and twelve staff members were also invited to participate. The age range of The Grafters Club members at the party was one to eighteen; siblings were also present with an age range of one year old to eighteen years old. Informed consent was assumed if participants chose to respond. Participants were seated within their family groups and were free to discuss their answers as permitted within the methodology. 

### 2.2. Methods

Group concept mapping was undertaken in accordance with methods described by Trochim and Kane [13] and using the GroupWisdom programme. In the brainstorming stage undertaken at the Christmas party, participants were given two sentence openings and asked to complete the sentences in their own words. 

Sentence 1: The challenges facing children and families after a burn injury are…

Sentence 2: The role of The Grafters Club is…

The participants were instructed to include only one point per response but that they could write as many responses as they wanted, and that they could complete one or both sentences. The participants were guided to write their responses on sticky-notes and then add to communal sheets designated for this purpose and displayed in the venue. Following completion of the brainstorming, sorting of the statements was undertaken. To complete this sorting, the responses were inputted into the GroupWisdom programme where they were reviewed by three of the staff respondents and organised into groups based on themes and similarities in meaning. The statements could only be placed in one group, and there could not be a group of miscellaneous statements. Once this was completed, the groups of statements were named according to their content. After this, nine staff members (selected due to having experience in attending Grafters Club activities) and one ex-member of The Grafters Club rated each individual statement on a scale of zero to ten based on how much they considered The Grafters Club to be successful at achieving this statement, and how important it is to the recovery and rehabilitation of children and families after a burn injury.

### 2.3. Statistical Analysis

A ‘point cluster map’ was generated from the sorting of the statements using multidimensional scaling analysis (representing the dis/similarity data as Euclidean distances on the x and y axes) and hierarchical cluster analysis (to partition the map hierarchically into non-overlapping clusters). A pattern match or ‘ladder graph’ was created from the importance/success ratings; the ratings were aggregated according to cluster and presented on side-by-side up/down axes to compare average cluster ratings and agreement between the two axes. Finally, a ‘go-zone’ plot was used to visually present areas for service improvement; this is a bivariate plot divided into quadrants based on average ratings of the clusters according to success and importance at a statement level. The resulting analysis and visual charts were presented back to a selection of the participants to discuss the findings.

## 3. Results

### 3.1. Sample

It is not known exactly how many of the attendees participated in the evaluation, but review of the handwritten notes suggests contributions from a broad range of individuals including from both children and parents. Forty-eight individual statements resulted from sentence one (challenges after a burn) and ninety-eight individual statements were generated in response to sentence two (role of the club). 

### 3.2. Results of Statistical Analysis

Table 1 shows the clusters resulting from the cluster analysis of the statements associated with each sentence. An eight-factor solution was selected for sentence one, and nine-factor solution for sentence two.

**Figure 1:**
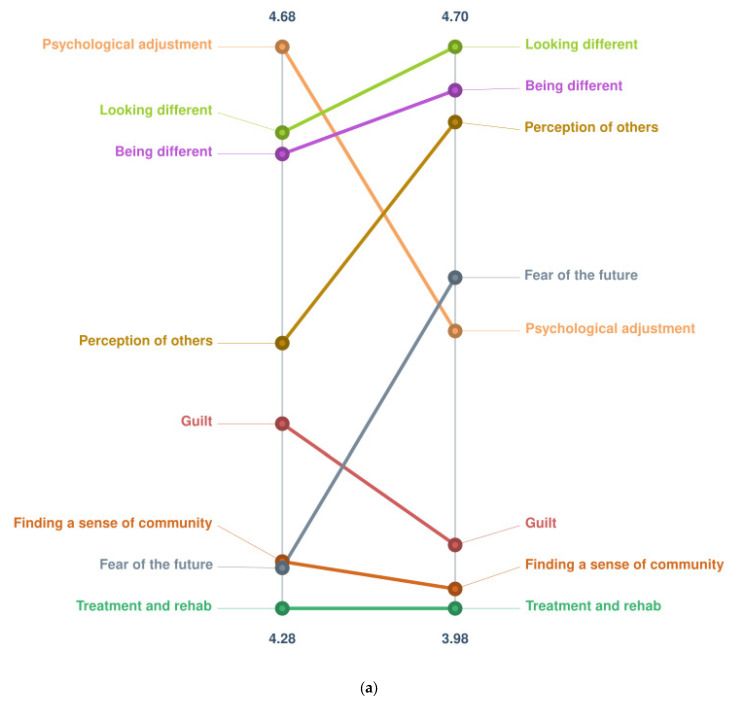

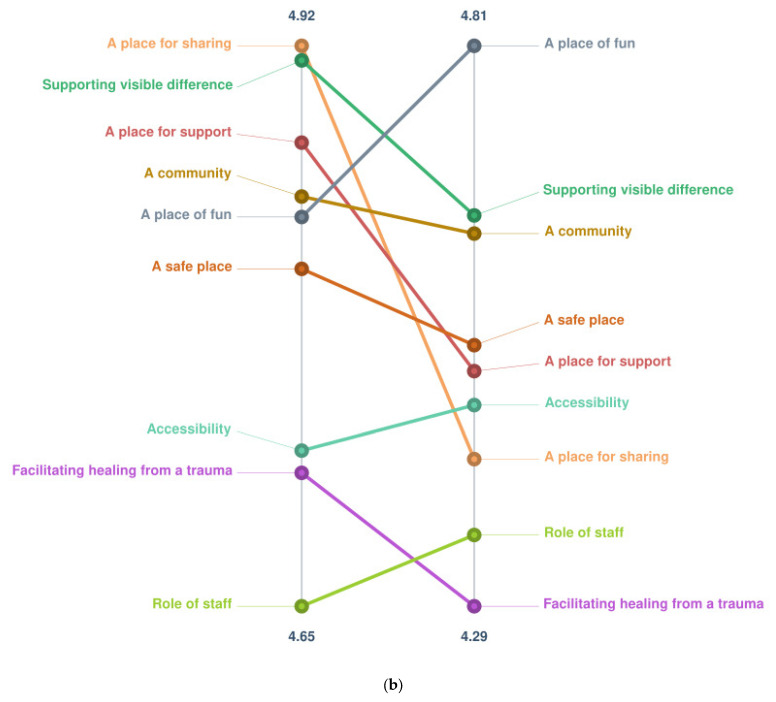
(**a**) Pattern graph for sentence one (The challenges facing children and families after a burn injury are…); **left hand axis**—importance ratings, **right hand axis**—success ratings. (**b**) Pattern graph for sentence 2 (The role of The Grafters Club is…; **left hand axis**—importance ratings, **right hand axis**—success ratings).

Figure 1a,b depict the pattern graphs for each sentence; the aggregated ratings of importance and success for each cluster are presented side by side to demonstrate the relationship between importance and success. The key points from Figure 1a (sentence 1: challenges after a burn) indicates that participants perceived that The Grafters Club was proportionately less successful at achieving “Psychological adjustment” (Importance 4.68 vs. Success 4.33), “Guilt” (I 4.41 vs. S 4.06) and “Sense of community” (I 4.31 vs. S 4.00) than their rated importance. It also suggests that it was proportionately more successful at achieving “Looking different” (I 4.62 vs. S 4.70), “Being different” (I 4.60 vs. S 4.64), “Perception of others” (I 4.47 vs. S 4.60) and “Fear of the future” (I 4.31 vs. S 4.40) than their importance. 

Figure 1b (sentence 2: role of the club) indicates that participants perceived that The Grafters Club was proportionately less successful at achieving “A place for sharing” (I 4.92 vs. S 4.42), “Supporting visible difference” (I 4.91 vs. S 4.65), “A place for support” (I 4.87 vs. S 4.50), “A community” (I 4.85 vs. S 4.63), “A safe place” (I 4.81 vs. S 4.53) and “Facilitating healing from a trauma” (I 4.71 vs. S 4.29) than their rated importance. Figure 1b also shows that participants perceived that it was proportionately more successful at achieving “A place for fun” (I 4.84 vs. S 4.81), “Accessibility” (I 4.73 vs. S 4.47) and “Role of staff” (I 4.65 vs. S 4.35) than their importance. 

**Figure 2:**
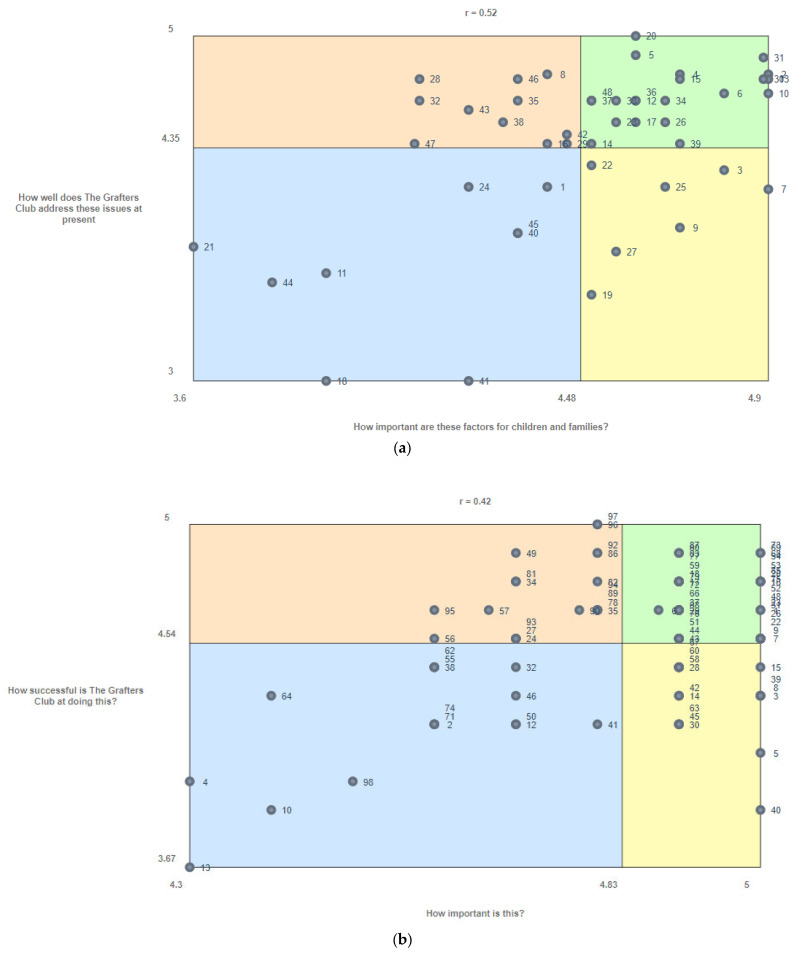
(**a**) Go-zone plot for sentence one (The challenges facing children and families after a burn injury are…). (**b**) Go-zone plot for sentence two (The role of The Grafters Club is…).

The ‘go-zone’ plots for sentence 1 (challenges after a burn; Figure 2a) and sentence 2 (role of the club; Figure 2b) suggests key areas to be targeted in service development at the level of the individual statements (the points contained within the yellow quadrant). Table 2 features the individual statements falling in this quadrant for each sentence.

### 3.3. Themes in the Data

In order to facilitate the interpretation and discussion of the results, the findings are discussed in terms of themes presented across both opening sentences. These themes were generated collaboratively by the authors directly based on the concepts yielded in Figure 1a,b and the specific responses highlighted in Figure 2a,b.

#### 3.3.1. Inclusion of the Whole Family

A number of the statements generated concerned “parental guilt”, were framed as parents citing a challenge in “helping the child…” live with the burn or coping with others’ reactions, or reflected a parental need to speak to other parents. The participants’ responses indicated an expectation for parental support from The Grafters Club rather than just support for the child with the burn injury. Many of the clusters generated from the data describe challenges particular to parents such as guilt or an awareness of the perception of others, and there were many statements that highlighted the role of The Grafters Club in terms of parents sharing their experiences with others. The results also indicate that the participants felt that The Grafters Club did not always meet these expectations, and that there was perhaps a gap between the perceived challenges faced by parents or the wider family and the support offered by the club. 

#### 3.3.2. Body Image and Scarring

It is clear from the results that participants recognised the challenges inherent in living with an altered body image due to scarring from a burn injury both because of their perception of their own/their child’s appearance and the awareness of the perception of others. The responses to sentence one (challenges after a burn) suggest that participants viewed The Grafters Club as successful at addressing these appearance-related issues, although this was less clear in the broader “visible difference” cluster from the results of sentence two (role of the club).

#### 3.3.3. Psychological Adjustment, Sharing Experiences and Building a Sense of Community

The go-zone plot for sentence two (role of the club) overwhelmingly highlights the need for the club to focus on the creation of a sense of community and providing opportunities for parents to share their lived experiences with each other. While participants did not necessarily highlight identifying a sense of community as a challenge after a burn relative to the other clusters, it was clearly recognised as part of The Grafters Club remit and something that is not currently being achieved. Alongside this, psychological adjustment (comprising statements including terms such as anxiety, depression and trauma) and guilt were both noted to be significant challenges following a burn injury but again something that was not achieved by the club’s current activities and events.

## 4. Discussion

The findings in this service evaluation reflect existing themes in the wider literature base for this population. Burn injuries can be traumatic and can trigger complex psychological reactions for both the child and their family including trauma responses, anxiety, low mood, body image distress, and guilt [7,14]. Families communicate a need to be considered as a whole unit by the burns service [15], and there appears to be an appetite for support that includes sharing stories with those who have lived experience of life after a burn injury [16]. 

The psychological concept of a “sense of community” [17] has been described as a dependable and mutually supportive network comprising the integration of four key factors: membership (a sense of belonging), influence (influencing others and being influenced oneself), integration and fulfilment of needs (a confidence that needs will be met through the community) and shared emotional connection (commitment to a belief that members have shared, and will continue to share, common experiences) [18]. Given the physical and psychological isolation reported by families following a burn injury, it is understandable that peer support systems would form this community. Historically, peer support in healthcare was initiated to create a cultural context of healing and recovery to promote self-expansion and a capacity for change following perceived reductionist medicalisation through a standard healthcare model [19]. For children and families living after a burn injury, peer support may create a sense of safety and normalisation that validates their experiences, supports them to process their grief/adjustment and trauma reactions, and move beyond the aftermath of the burn injury to a ‘new normal’ of life alongside it. 

Both from the literature base and past Grafters Club activity, there is a definite appetite for peer support amongst parents and yet attendance is often poor and parents cite multiple barriers [8,10]. Within the paediatric burn population, it is noted that, pre-injury, there are elevated rates of parental mental health difficulties and childhood behaviour disorders, as well as fewer years of parental education and a lower socioeconomic status than the non-burned population [20,21,22,23]. Perhaps these factors predispose families to be less willing or less able to attend formal psychological support either due to practical factors like finance or childcare, or they may have a negative perception of formal psychological therapy. It is considered then that perhaps less formal psychosocial care, as in the supportive conversations that happen between parents at burn club events, may be more appealing and less likely to trigger the connotations of accessing psychological therapy and therefore act as a vehicle to normalise parents’ reactions and promote adaptive grieving and adjustment to the injury.

The BBA Burn Care Standards [2] state that access to a burn camp or club is an essential component of burn care for children and families. Findings from this evaluation support this standard, but attention should be given to the wider service structure and provision when planning camp/club activities. Services with access to a robust and well-attended parental peer support service may be able to focus on primarily offering a traditional burn camp model to specifically address the child’s psychological needs around body confidence and self-esteem. Those services without separate parental peer support should consider whether a club model with accessible events for the whole family would be useful. In both of these settings, this should be distinct from and work parallel to formal psychological screening or intervention, and with closely connected multidisciplinary working between outpatient services such as scar management, physiotherapy, occupational therapy, nursing and clinical psychology. When planning camp and club events, consideration should be given to the skill mix of the staff ensuring either a clinical psychologist is present or alternatively an experienced burn care professional confident in facilitating conversations between parents in order to promote story sharing and psychological processing. Events such as preschool soft play or music/dance sessions with parent refreshments could present an ideal balance between encouraging attendance with a child-focused activity and promoting the sharing of lived experiences amongst parents.

### Limitations

The limitations to the findings from this study should be considered. The results from the statistical analysis all demonstrate a very narrow range of rated importance between 3.98 and 4.92, indicating a possible response bias and ceiling effect within the data. All the participants included in the group mapping were personally or professionally invested in The Grafters Club, and desirability bias may be particularly relevant for parent/child responses. The Group Concept Mapping methodology permits the use of a broad and varying population, and hence the conflation of statements from staff, children and parents. However, this does make it impossible to differentiate between the perspectives of these groups. Future research should consider using either alternative methodologies or group concept mapping with the separate groups to delineate between their perspectives. While the sample size used at each stage in the process was appropriate for the methodology, the sub-sample used in the sorting and rating stages was heavily weighted towards Grafters Club staff due to accessibility to the parent/child participants having utilised a convenience sample for the initial data generation. For these reasons, wider exploration of patient experience evaluation of burn services indicates that there should be a move away from single-source quantitative methodologies to a triangulation approach utilising quantitative methods, staff feedback, service and national priorities, focus groups, and patient stories (unpublished activity from the National Patient Experience Working Group, UK). Future service development of burn camps and clubs should explore this triangulation approach to increase the clarity of detail in specific areas of concern to form a more comprehensive evaluation.

## 5. Conclusions 

The results of this evaluation support previous findings in the literature that burn camps and club activities are perceived to have significant benefit for children living with a burn injury in terms of adjusting to a changed body image and developing body confidence when living with a scar, and reflections on themes of psychological healing and recovery reflect wider findings around the impact of burn injuries on children and their families. However, this evaluation also demonstrates that burn clubs are perceived to have a much broader role in facilitating the complex psychological healing and recovery not just of the child but of the family and wider support network. Burn clubs should offer events that permit the coming together of parents to share their stories and experiences in an informal setting, and therefore should ensure that those staffing these events are experienced in facilitating these discussions. 

## Figures and Tables

**Table 1:** Clusters identified for each sentence prompt.

Sentence 1: Challenges after a Burn	Sentence 2: Role of the Club
Guilt	A place for support
Looking different	The role of staff
Psychological adjustment	A place for sharing
Treatment and rehabilitation	Supporting visible difference
Fear of the future	A place for fun
The perception of others	A community
Being different	Facilitating healing from a trauma
Finding a sense of community	A safe place
	Accessibility

**Table 2:** Individual statements identified as an area of need within the go-zone plots for sentences one and two.

Sentence 1: Challenges after a Burn	Sentence 2: Role of the Club	
3 overcoming daily physical challenges	39 a safe place to be and share experiences	40 accompanying and supporting families
7 trauma reactions	5 parents supporting each other	8 sharing experiences
19 not able to do normal stuff	42 learn to overcome traumatic experiences	14 opportunities for both child and parent to speak with others in similar situations
22 feeling guilty	45 to create a community for burn victims and their family	
25 feelings of guilt		
27 avoidance of further injury	3 opportunity for children and families to speak to others in similar situations	63 parents have the opportunity to discuss with other parents

## Data Availability

Restrictions apply to the availability of these data due to consent and confidentiality of the clinical population used for the study.
